# Immunotactoid glomerulopathy in rheumatoid arthritis 

**DOI:** 10.5414/CNCS111805

**Published:** 2025-12-11

**Authors:** Adriana Dejman, Jaylou M. Velez Torres, Youley Tjendra, Juan C. Duque, Yiqin Zuo

**Affiliations:** 1 Katz Division of Nephrology, Department of Medicine, and; 2 Department of Pathology and Laboratory Medicine, Miller School of Medicine, University of Miami Hospital, Miami, Florida, USA

**Keywords:** immunotactoid glomerulopathy, rheumatoid arthritis, IgG subclasses

## Abstract

In this clinical case report, we explore a rare type of renal involvement in a patient with rheumatoid arthritis: the polyclonal variant of immunotactoid glomerulopathy. To date, 26 cases of this rare variant have been reported, and our patient is the second solely associated with rheumatoid arthritis. Our report includes a detailed case description, follow-up, and an extensive review of immunotactoid glomerulopathy with comprehensive differential diagnostic considerations.

## Introduction 

Organized microtubular or fibrillary glomerular deposits under ultrastructural assessment occur in a variety of renal diseases, including amyloidosis, cryoglobulinemic glomerulonephritis, lupus nephritis, fibrillary glomerulonephritis (FGN), and immunotactoid glomerulopathy (ITG). ITG is rare but is characterized by distinctly different substructures of the deposits, with monoclonal and polyclonal variants. To date, 26 cases of the polyclonal variant have been reported. Among these, 10 reportedly had an autoimmune disease, and 1 additional case had an infectious condition. This case report presents the clinical presentation, renal biopsy findings, additional immunofluorescence for IgG subclasses, and follow-up of a patient with rheumatoid arthritis (RA) diagnosed with polyclonal ITG. 

## Case report 

This study was approved by the Institutional Review Board of the University of Miami. 

A 50-year-old white Hispanic woman was admitted for worsening renal function and underwent a renal biopsy. Her medical records were reviewed, including history, medications, laboratory results, and imaging. The biopsy was processed using standard techniques: light microscopy (LM), immunofluorescence (IF), and electron microscopy (EM). For LM, 2- to 3-µm sections were stained with hematoxylin and eosin, periodic acid Schiff (PAS), and Jones’ methenamine silver. For IF, 4-µm cryostat sections were stained with polyclonal fluorescein isothiocyanate (FITC)-conjugated antibodies to IgG, IgA, IgM, C3, C1q, κ and λ light chains, fibrinogen, and albumin, with additional IgG subclasses testing. For EM, thick sections were stained with toluidine blue, thin sections were cut, and digital images were captured using a JOEL JEM-1400 transmission electron microscope. IF results and EM images were evaluated by a renal pathologist. 

### Patient presentation 

The patient has over 12 years of documented history of RA but never followed up with a rheumatologist or used any disease-modifying antirheumatic drugs (DMARDs) or biologic drugs. She managed pain with daily nonsteroidal anti-inflammatory drugs. She presented with worsening lower extremity edema, shortness of breath and uncontrolled hypertension (188/110 mmHg). Physical examination revealed acute synovitis in the left elbow, second to third metacarpophalangeal joints, and bogginess over both knees, with no rashes or vasculitis. Lab results showed impaired renal function (serum creatinine up to 3.5 mg/dL in 11/2020, 2.2 mg/dL in 6/2020, 0.85 mg/dL in 5/2019), hypoalbuminemia (2.0 g/dL), positive rheumatoid factor (140 IU/mL), cyclic citrullinate peptide IgG (30.4 units/mL), actin IgG anti-smooth muscle, and anti-mitochondrial M2 antibodies. Additional workup showed a urine protein-to-creatinine ratio (UPCR) 12.3 g/g, active urine sediment (92 RBC/high power field and 44 WBC/high power field), negative anti-dsDNA, anti-Smith, anti-RNP antibodies, ANCA, cryoglobulin, HIV, and hepatitis B and C, normal complement levels and serum free light chain ratio, and no monoclonal bands detected. 

## Results 

### Kidney biopsy findings 

**Figure 1:**
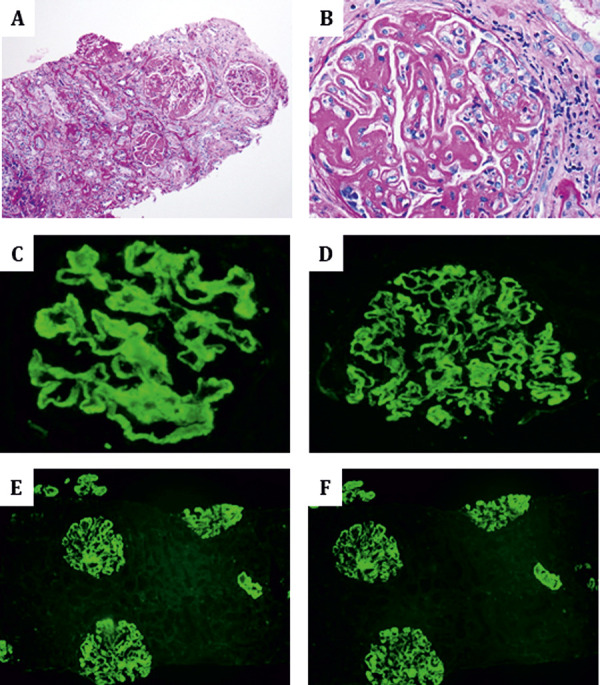
Light microscopy and immunofluorescence findings: A – B: mesangial expansion by eosinophilic material and increased cellularity, thickening and splitting of glomerular basement membranes and endocapillary hypercellularity (PAS stain, A: 100 ×; B: 400 ×). C – F: Smudgy and/or chunky staining along the capillary loops with segmental mesangial extension for IgG, C3, and kappa and lambda light chains (C: IgG, 400 ×; D: C3: 400 ×; E: kappa: 100 ×; F: lambda: 100 ×).

**Figure 2:**
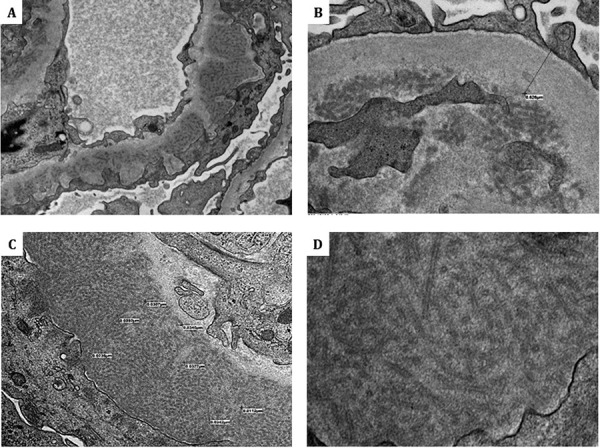
Electron microscopy findings: numerous subepithelial deposits and occasional subendothelial deposits exhibiting a microtubular substructure with hollow cores arranged focally in parallel arrays and measuring 11 – 72 nm with an average of 34 nm (A: 4,000 ×; B: 10,000 ×; C: 8,000 ×; D: 12,000 ×).

The renal biopsy showed 15 glomeruli, none globally sclerosed, and ~ 20% interstitial fibrosis. LM revealed mesangial expansion by eosinophilic material and increased cellularity, diffuse thickening and segmental splitting of glomerular basement membranes, and segmental endocapillary hypercellularity (Figure 1). IF showed 3+, diffuse, segmental to global, smudgy and/or chunky staining along the capillary loops with segmental mesangial extension for IgG, C3, and κ and λ light chains with lesser IgM and C1q (Figure 1). EM showed numerous subepithelial deposits and occasional subendothelial deposits, which exhibit a microtubular substructure with hollow cores arranged focally in parallel arrays and measuring 11 – 72 nm with an average of 34 nm (Figure 2). A Congo red stain was negative. The diagnosis of ITG, a polyclonal variant, was rendered. 

### Additional testing and follow-up 

**Figure 3:**
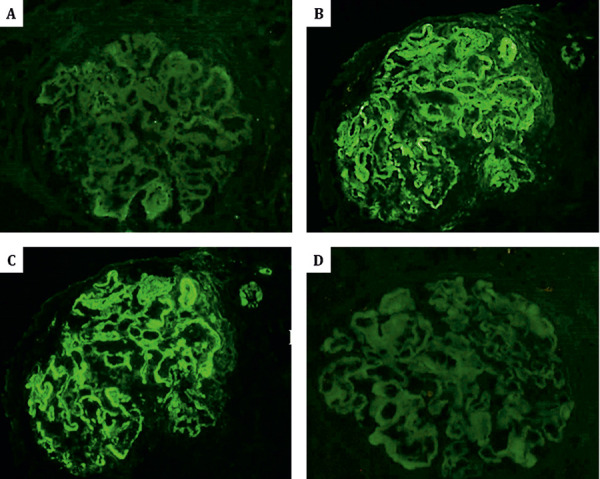
Immunofluorescence for IgG subclasses: positive for IgG2 and IgG3 with the same pattern as IgG, and negative for IgG1 and IgG4 (all 400 ×, A: IgG1; B: IgG2; C: IgG3; D: IgG4).

Since the polyclonal variant of ITG is rare, IgG subclasses testing was conducted on the IF specimen. It showed positivity for IgG2 and IgG3 with the same pattern as IgG, while negative for IgG1 and IgG4 (Figure 3), confirming the polyclonal composition. 

Further workup did not show evidence of hematologic disorders following the renal biopsy. The patient’s serum creatine was 2.1 mg/dL at discharge in November 2020. The patient received intermittent follow-up care from a rheumatologist, nephrologist, and internal medicine physician. She was considered unsuitable for methotrexate or biological drugs and was instead prescribed prednisone, mycophenolate, and anti-hypertensive medications with adjusted dosages. Due to financial constraints, she was not treated with rituximab. She was not fully compliant with the treatment. Initially, within 15 months post-biopsy, her renal function improved, with serum creatinine levels decreasing to 1.1 – 1.6 mg/dL. Her cyclic citrullinate peptide IgG dropped to 22.7 units/mL, while her rheumatoid factor level increased to 223 IU/mL. Despite this, her proteinuria remained in the nephrotic range (UPCR: 3.5 – 8.9 g/g), and her blood pressure was poorly controlled. Two years post-biopsy, her serum creatinine increased to 2.1 mg/dL, rapidly rising to 6.8 mg/dL within 3 months. She has been on dialysis ever since. 

## Discussion 

ITG, is an extremely rare disease, accounting for 0.06% of adult native kidney biopsies [1]. The diagnosis relies on the ultrastructural assessment of kidney biopsies, revealing glomerular deposits with parallel arrays of microtubular substructure, usually over 30 nm in diameter, with a distinct hollow core [2, 3]. Light microscopy findings are varied, and immunofluorescence studies are typically positive for IgG and C3, often with light chain restriction. Two recent studies have identified light chain-only ITG, with one group recognizing κ-only and the other λ-only [4, 5]. ITG is subclassified into monoclonal and polyclonal variants based on κ and λ light chains and IgG subclasses by IF [6]. 

Clinically, patients with ITG present with proteinuria, hematuria, hypertension, and renal insufficiency. ITG is often associated with indolent or malignant lymphocytic or plasma cell disorders, especially chronic lymphocytic leukemia [7, 8]. Not surprisingly, the monoclonal variant ITG has a higher incidence of hematologic diseases. Given its association with indolent hematologic diseases and/or monoclonal gammopathy of undetermined significance (MGUS), ITG is included among the spectrum of monoclonal gammopathy of renal significance (MGRS)-defining lesions [9, 10]. Recent studies have shown that ITG coexists with monoclonal immunoglobulin deposition disease [11, 12]. 

Due to its rarity, literature on ITG is limited to case reports and small case series [1, 13, 14]. In 2021, Nasr et al. [6] reported the largest series of 73 cases of ITG in a retrospective study involving two large renal biopsy referral centers in the United States from 1993 to 2019, describing both monoclonal and polyclonal variants of ITG. Javaugue et al. [7] reported on 27 adults with monoclonal ITG from 21 French nephrology centers from 1980 to 2018. 

The monoclonal variant ITG has been extensively studied. In Nasr et al.’s study, it accounted for 67% of cases (49 of 73). Patients who underwent clone-directed therapy showed good primary renal outcomes, measured by the occurrence of end-stage kidney disease (ESKD) or death [6]. The French study on the monoclonal variant investigated renal and hematologic outcomes, defining hematologic outcomes based on clonality, and found that early detection and hematologic response after clone-targeted chemotherapy were linked to favorable outcomes [7]. In Nasr et al.’s series, the hematologic diagnosis predated the kidney biopsy in 50% of cases, compared to only 19% in the French series. Recent case reports of monoclonal ITG related to lymphomas have shown similar results with better prognoses [15, 16, 17]. Overall, substantial evidence highlights the importance of a thorough pathologic and hematologic workup in managing ITG. 

In comparison, the polyclonal variant of ITG is even rarer, with only 26 reported cases, including 24 patients in Nasr et al.’s series [6, 18, 19]. Among these 24 patients, 8 had positive ANA, and 10 had an underlying autoimmune disease, including 1 with RA and 1 with both lupus and RA. Interestingly, the incidence of hematologic conditions was higher than expected in the general population, with 2 lymphoma, 3 MGUS, and 2 with abnormal serum free light chain ratio. 17 patients were followed up and treated with supportive care, steroid only, or steroid with another immunosuppressant. Over a mean follow-up of 48 months, 29% died, and 53% reached ESKD. Four patients with repeated biopsies still showed the polyclonal pattern [6]. Additionally, Shimizu et al. [19] reported a patient with a 14-year history of nontuberculous mycobacterial infection whose renal biopsy showed a polyclonal ITG; this patient showed decreased proteinuria and slowed renal function decline after receiving an additional antibacterial agent. Another patient with polyclonal ITG associated with IgM κ-type MGUS treated with rituximab alone showed stable renal function and decreased proteinuria over 32 months [18]. 

Our patient has a history of RA and has not been treated with DMARDs or biologic drugs for over 10 years. RA, an autoimmune disease, primarily affects synovial joints but can also have extra-articular manifestations, including kidney involvement. Renal involvement in RA can result from the disease itself or secondary to the medications used for treatment. Differential diagnoses include amyloid A (AA) amyloidosis, mesangial proliferative glomerulonephritis, membranous nephropathy, and IgA nephropathy [20, 21]. Our patient’s biopsy showed mesangial expansion by eosinophilic material and a negative Congo red special stain, ruling out amyloidosis. IF showed IgG and C3 co-dominant staining with equal κ and λ light chains, along with IgG2 and IgG3 positivity, confirming the polyclonal nature of the deposits. EM demonstrated deposits with organized substructures, bringing additional differential diagnostic considerations, including lupus nephritis, cryoglobulinemic glomerulonephritis, and FGN. There was no clinical suspicion of lupus. Negative anti-dsDNA, anti-Smith antibodies, and no full-house staining by IF ruled out lupus nephritis. Physical examination showed no rashes or vasculitis. Laboratory results revealed a negative serological workup for hepatitis B and C, negative cryoglobulin, and normal complement levels. No PAS-positive cryo-plugs were identified by LM. There was lesser IgM, without κ/λ shift by IF, and no annular or cylindrical substructures within deposits by EM, arguing against cryoglobulinemic glomerulonephritis. FGN typically shows randomly arranged fibrils ranging from 9 to 25 nm, averaging 15 nm in diameter, without a hollow core by EM and an IgG4-dominant pattern by IF. Notably, in Nasr et al.’s series, 23 specimens with microtubules measuring 20 nm or less in diameter were further tested for DNAJB9, a highly specific marker for FGN, and all were negative. In our case, the deposits exhibited a microtubular substructure with hollow cores arranged focally in parallel arrays with an average diameter of 34 nm by EM, and there was IgG2 and IgG3 positivity without IgG1 or IgG4 by IF, arguing against the diagnosis of FGN. Given the patient’s financial situation, DNAJB9 testing was not further performed. Our findings indicated the diagnosis of polyclonal ITG associated with RA. During follow-up, our patient initially responded well within 15 months post-biopsy but unfortunately progressed to ESKD 2 years post-biopsy, partially due to poorly controlled underlying disease and long-term steroid usage. 

The pathogenesis of ITG and the biochemical mechanisms underlying microtubule formation remain incompletely understood. While immune complex deposition is common to both autoimmune-associated glomerulonephritis and polyclonal ITG, our case highlights the unusual presence of organized microtubular substructure in a setting of systemic autoimmunity, supporting the view that ITG represents a distinct pathophysiologic entity. In autoimmune glomerulonephritis, deposits are typically amorphous and granular, though rare cases – particularly in cryoglobulinemic glomerulonephritis or lupus nephritis – may exhibit organized curved, microtubular, curvilinear, or fingerprint-like substructures. Whether this organization reflects a morphologic variant or a different underlying mechanism remains uncertain. The presence of microtubular substructure in ITG suggests altered physicochemical properties of immunoglobulins, potentially due to conformational or glycosylation changes, that promote self-aggregation – even in polyclonal cases. Although both polyclonal and monoclonal ITG may share similar ultrastructures, the drivers of this organization remain unknown. Our case underscores the diagnostic value of EM in autoimmune glomerulonephritis, as the identification of substructure may point to a distinct pathogenic process and prompt further evaluation for underlying immune dysregulation or paraproteinemia. Additional studies are needed to clarify the mechanisms responsible for this unique pattern of immune complex organization. 

In summary, ITG is a rare disease requiring accurate diagnosis, subclassification, and thorough evaluation to identify underlying conditions and determine the appropriate treatment. 

## Authors’ contributions 

A.D.: manuscript preparation, data analysis, and interpretation. J.V.T.: manuscript and figure preparation. Y.T.: manuscript and figure preparation. J.C. D: manuscript and figure preparation, patient follow up. Y.Z.: original idea, manuscript preparation, data analysis, and interpretation. 

## Funding 

The authors received no financial support for this article’s research, authorship, and/or publication. 

## Conflict of interest 

The authors declared no potential conflicts of interest concerning this article’s research, authorship, and/or publication.
